# The impact of immune response on endochondral bone regeneration

**DOI:** 10.1038/s41536-018-0060-5

**Published:** 2018-11-29

**Authors:** A. Longoni, L. Knežević, K. Schepers, H. Weinans, A. J. W. P. Rosenberg, D. Gawlitta

**Affiliations:** 1 Department of Oral and Maxillofacial Surgery & Special Dental Care, University Medical Center Utrecht, Utrecht University, Utrecht, G05.222, PO Box 85500, 3508 GA The Netherlands; 2 Regenerative Medicine Center Utrecht, 3584 CT Utrecht, The Netherlands; 3 Faculty of Health Sciences, University of Bristol, Biomedical Sciences Building, Bristol, BS8 1TD UK; 4 Department of Immunohematology and Blood Transfusion, Leiden University Medical Center, 2300RC Leiden, The Netherlands; 5 Department of Orthopaedics, University Medical Center Utrecht, Utrecht University, 3508 GA Utrecht, The Netherlands; 6 Department of Rheumatology, University Medical Center Utrecht, Utrecht University, 3584CX Utrecht, The Netherlands; 7 Department of Biomechanical Engineering, Delft University of Technology, 2628CD Delft, The Netherlands

## Abstract

Tissue engineered cartilage substitutes, which induce the process of endochondral ossification, represent a regenerative strategy for bone defect healing. Such constructs typically consist of multipotent mesenchymal stromal cells (MSCs) forming a cartilage template in vitro, which can be implanted to stimulate bone formation in vivo. The use of MSCs of allogeneic origin could potentially improve the clinical utility of the tissue engineered cartilage constructs in three ways. First, ready-to-use construct availability can speed up the treatment process. Second, MSCs derived and expanded from a single donor could be applied to treat several patients and thus the costs of the medical interventions would decrease. Finally, it would allow more control over the quality of the MSC chondrogenic differentiation. However, even though the envisaged clinical use of allogeneic cell sources for bone regeneration is advantageous, their immunogenicity poses a significant obstacle to their clinical application. The aim of this review is to increase the awareness of the role played by immune cells during endochondral ossification, and in particular during regenerative strategies when the immune response is altered by the presence of implanted biomaterials and/or cells. More specifically, we focus on how this balance between immune response and bone regeneration is affected by the implantation of a cartilaginous tissue engineered construct of allogeneic origin.

## Introduction

Bone healing is a remarkable process that can deliver fully functional and integrated new tissue, without scar formation.^1^ Due to this regenerative capacity, the majority of bone fractures, which are the most common large organ injuries, reach resolution through complete healing. Nevertheless, 10% of all fractures do not completely heal, resulting in failed bridging of the bone defect, called a non-union.^2^ In addition, certain bone degenerative disorders, as well as osteosarcomas, can result in loss of bone tissue that cannot be repaired through the natural healing process.^1^ Bone grafting has been the treatment of choice in such cases, primarily autologous, and occasionally allogeneic. However, both options have well-known disadvantages: the first one includes morbidity of the surgical site from where the graft is removed, while the latter bares the risks of immune rejection and disease transmission.^3^ Besides, the scarcity of graft material represents another driving force behind the search for alternatives.^3^

Tissue engineered bone constructs represent an attractive alternative. Traditionally, they rely on osteogenic cells seeded in 3D scaffolds to enhance the natural healing capacity of the recipient.^4^ The most commonly employed regenerative strategy is to mimic the intramembranous repair process, where a bone matrix is directly synthesized in vitro and subsequently implanted in vivo.^4,5^ So far, these cell-seeded constructs have shown greater potential in vitro compared to in vivo, probably due to insufficient vascularization of the constructs upon implantation.^4,6^ A promising alternative strategy exploits the chondrogenic potential of cells to mimic the endochondral ossification process. Similarly to the long bone natural development, during the tissue regeneration therapies, an implanted cartilaginous template will acquire a hypertrophic chondrogenic phenotype; will be invaded by blood vessels, host osteoblasts and osteoclasts, and will eventually be converted into bone tissue.^4,5,7,8^ The endochondral strategy encompasses several advantages over other cell-based approaches. For example, chondrocytes can survive in low-nutrient environments,^5,9^ and are thus an attractive cell source for implantation. Also, this eliminates the need for an integrated vascular network, simplifying the culturing process.^6^ Further, the proposed terminal nature of the hypertrophic chondrocyte differentiation^2,10^ suggests an eventual deletion of the majority of the implanted cells.^11^ These features together with the robustness and efficiency of this approach^7,11–15^ make endochondral bone regeneration (EBR) an appealing strategy for clinical translation.

However, some considerations pertain to the clinical translatability of the approach. Currently, bone-marrow-derived multipotent mesenchymal stromal cells (MSCs) are the most frequently used cell source for EBR research.^4^ Although adipose-derived stem cells may be an alternative cell source for EBR,^16,17^ only few reports exist to date. Thus, in this review we focus on bone-marrow-derived MSCs. MSCs are not only capable of differentiating toward the chondrogenic lineage,^18^ but they also spontaneously progress into a hypertrophic phenotype,^19^ which is a particularly favorable characteristic for the endochondral application. However, the development of bone substitutes using MSCs requires expansion and in vitro differentiation to produce an implantable cartilaginous template. The (1) unpredictable lengthiness of the pre-operative laboratory work, which includes MSC isolation, expansion, characterization, and differentiation; together with (2) the difficulties in synchronizing the process with the surgical schedule; and most importantly, (3) the heterogeneity in differentiation potential between MSCs isolated from different donors,^13,20^ pose an obstacle for the use of autologous MSCs and the second point also for allogeneic MSCs. Furthermore, the harvest of autologous cells represents an additional discomfort for the patient and a logistical challenge, as it involves an invasive intervention for the patient prior to the regular operation for bone reconstructive purposes. Finally, high costs are associated with growing and differentiating the MSCs under Good Manufacturing Practice conditions when performing such a procedure in a personalized fashion.^21^

Allogeneic cell sources represent an attractive alternative, offering the possibility of developing a “ready-to-use” product.^22^ In particular, allogeneic MSCs could be isolated, expanded, and characterized for their hypertrophic chondrogenic potential in advance, reducing the time required to produce the graft substitute, avoiding complex logistics and the need of two interventions for the patient. In addition, this approach would benefit patients whose own MSCs have a lower chondrogenic potential, such as the elderly.^23,24^ Lastly, MSCs harvested from one donor could be used to treat multiple patients, which would reduce the costs of treatment considerably. Obviously, the use of non-autologous cell sources in EBR could potentially simplify the implementation into the clinical practice. However, the main problem posed by the use of non-autologous cells is their immunogenicity. Transplanted cells could be recognized and cleared by the host immune system, preventing the integration and the remodeling of allogeneic tissue engineered constructs.^25,26^ Furthermore, it is known that an extensive crosstalk exists between bone cells and cells of the innate and adaptive immune systems during bone development and fracture healing.^27^ For instance, there is consistent evidence in literature of new bone formation enhancement achieved by promoting the initial acute inflammatory response with localized pro-inflammatory stimuli.^28–31^ However, altering the homeostasis between immune and bone cells by, for example, inducing a chronic inflammatory condition due to the presence of allogeneic cells, might negatively affect the balance between bone formation and resorption.^32^ This could lead to the failure of the EBR process. Apart from studies focusing on bone regeneration following implantation of allogeneic osteogenically differentiated MSCs,^26,33,34^ the in vivo regenerative potential of non-autologous MSCs has been studied mainly on non-differentiated MSCs^35–38^ or in immunocompromised animal models.^4,7,15^ Thus, the role of the immune system in EBR, in particular when allogeneic, chondrogenically differentiated MSCs will be used, is largely unknown.

The scope of this review is to highlight the immunological aspects that can affect the outcome of EBR strategies. To this end, a general analysis of the role of the immune system in endochondral fracture healing and in response to implanted cells and/or biomaterials is provided. Then EBR is detailed before we propose a speculative analysis of the feasibility of using allogeneic, chondrogenically differentiated MSCs for EBR. Further, understanding the fate of the allogeneic chondrocytes after implantation will help elucidating if the exposure to allogeneic epitopes is only a transient or long-lasting challenge for the host immune system.

## The role of the immune system in bone homeostasis and healing

Two distinct bone forming processes are responsible for fracture healing, namely intramembranous and endochondral ossification. Intramembranous ossification, which involves the direct differentiation of MSCs into osteoblastic cells, is mainly found in bone healing of fractures characterized by high mechanical stability due to the presence of, for example, rigid fixation.^2,8^ On the other hand, the healing of larger defects with mechanical instability due to macro and micromotion between the bone edges (e.g., fractures treated in a cast or with traction) occurs predominantly through endochondral ossification.^8^ In this section the cascade of events occurring during endochondral ossification will be reviewed together with the approaches used to mimic this process for regenerative purposes. Furthermore, the cells and factors from the innate and adaptive immune systems relevant in EBR will be presented. This will provide the basis to understand the cellular and molecular interactions of immune cells and cells involved in bone regeneration.

### Endochondral bone formation in fracture healing

After trauma, two areas are primarily involved in bone repair: at the periphery of the fracture site the periosteum elevation mediates direct bone deposition, whereas in the central region of the defect, a cartilaginous soft callus is formed in order to stabilize it.^8^ The structure of the fracture callus has often been compared to the one of the growth plate, present during long bone development. Both structures present an organized cartilaginous template composed of similar structural proteins (e.g., collagen types I, II, and X) and signaling molecules (e.g., Indian hedgehog, bone morphogenetic proteins).^8,39^ Also, the resident chondrocytes are arranged in a zonal fashion.^5^ In a first zone, chondrocytes are embedded in an avascular matrix, rich in collagen type II and proteoglycans. In the adjacent areas, chondrocytes proliferate and organize themselves into columnar structures, where they acquire a hypertrophic phenotype.^4,5^ Few changes mark the chondrocyte transition towards hypertrophy. Firstly, they start synthesizing collagen type X, metalloproteinases (e.g., MMP-2, MMP-9, and MMP-13) and proangiogenic factors, including transferrin and vascular endothelial growth factor (VEGF).^4,5^ Furthermore, chondrocytes undergo morphological changes, considerably increasing their size.^5,40^ Finally, this stage is characterized by a downregulation of genes involved in chondrogenesis followed by an upregulation of those involved in osteogenesis, including runt-related transcription factor 2, alkaline phosphatase and osteonectin, which will eventually lead to the mineralization of the cartilaginous matrix.^5,41^ The remodeling of the cartilaginous matrix, promoted by the presence of the degrading enzymes, metalloproteinases, in combination with the secretion of proangiogenic factors, facilitate blood vessel invasion and the infiltration of osteoprogenitor cells and osteoclasts.^4,10^ As a consequence, the mineralized cartilage matrix is replaced by woven bone to form a more stable hard callus.^42^ Finally, the woven bone is remodeled by the concerted actions of osteoblasts and osteoclasts, and the original cortical and/or trabecular bone architecture is restored.^39,42^

### EBR strategies

The feasibility of recapitulating the above described natural healing process for regenerative purposes has been widely explored in the last decades.^4,7,11–15,43,44^ Several studies demonstrated that in vitro engineered cartilage templates obtained from MSCs alone,^13,19,44^ or in combination with different biomaterials,^14,15,43,45^ could be successfully converted into new bone tissue upon implantation, both ectopically^12,19^ and orthotopically.^13,14,43,45^ However, so far no consensus has been reached regarding the optimal length of the period for chondrogenic differentiation prior to implantation.^4,46^ It could span from as little as 1 week^44^ to 7 weeks.^19^ Also, no agreement exists regarding the optimal differentiation status (chondrogenic or hypertrophic chondrogenic) before implantation.^4,46^ These issues were explored in a recent publication by Yang et al.,^47^ where the effect of different chondrogenic priming periods preceding implantation on endochondral bone formation was explored. In particular, when rat MSCs were chondrogenically differentiated for 2, 3, or 4 weeks, differences in glycosaminoglycans (GAG) content and extracellular matrix distribution were found prior to the in vivo implantation. Nevertheless, this did not lead to differences in bone volume after 8 weeks of subcutaneous implantation.^47^ This was explained by the fact that the markers, which are typical of the hypertrophic stage (VEGF and collagen type X), were already present in the constructs after 2 weeks. This indicates that, as soon as expression of factors related to the hypertrophic stage is reached, further differentiation in vitro may not be required to maximize the extent of new bone formation.

After implantation, one of the most interesting aspects to consider is the contribution of the donor (implanted) cells to the new bone formation. After 4–16 weeks, the newly formed bone tissue presents an appearance similar to native bone, with a cortical outline and an inner bone marrow-like structure.^11,12,14^ Scotti et al.^12^ determined the contribution of xenogeneic, chondrogenically differentiated MSCs to endochondral bone formation in a subcutaneous, immunodeficient mouse model by staining the explants for specific human *Alu* repeats. Interestingly, after 12 weeks, donor-derived cells were present in the more inner, trabecular-like bone structures. On the contrary, the outer, cortical-like bone was completely remodeled and populated by donor-derived cells.^12^ Comparable results were obtained by Farrell et al.^11^ and by Bahney et al.^48^ after the subcutaneous implantation of rat and human chondrogenically differentiated MSCs in a co-isogenic rat and immunodeficient mouse model, respectively. In particular, the presence of donor-derived osteocytes was confirmed after 6^48^ and 8 weeks^11^ of implantation, demonstrating the active contribution of the tissue engineered cartilaginous template to the endochondral ossification process. On a final note, implanted MSCs could be involved in recruiting host cells at the remodeling site, promoting neovascularization and new bone formation.^4^ Long-term persistence of implanted cells in the bone tissue has not been investigated to date. It is to be expected that depending on the size of the implanted construct, the natural process of bone remodeling will eventually replace the implanted cells with host cells.

### Key players of the immune system in bone healing

When bone is fractured, it usually results in damage of the surrounding tissues and vasculature, thereby inducing a state of inflammation and the formation of a hematoma.^49^ The hematoma environment is characterized by a low pH,^49^ hypoxia,^50^ high concentrations of both pro- and anti-inflammatory cytokines,^2,49,51,52^ and both innate and adaptive immune cells invading from the peripheral blood and the surrounding tissues.^49,51^ The first cells to act in the fracture zone are neutrophils^53^ that prevent the spread of pathogens and attract macrophages to the injured site.^51^ Following neutrophil infiltration, tissue resident macrophages, together with the infiltrating macrophages, release pro-inflammatory cytokines, and promote mesenchymal stem cell migration to the hematoma.^2,42,53^ Here, endogenous mesenchymal stem cells are directly involved in the fracture healing process. In particular, they can differentiate towards both, the chondrogenic lineage to participate in the synthesis of the cartilaginous matrix of the soft callus; and the osteogenic lineage to promote intramembranous ossification at the fracture edges.^4^ In response to the inflammatory environment, the infiltrating macrophages acquire a pro-inflammatory phenotype (M1), secreting pro-inflammatory cytokines, including interleukin-6 (IL-6), tumor necrosis factor-α (TNF-α), and interferon-γ (IFN-γ).^54^ This eventually leads to amplification of the pro-inflammatory response and to the activation of the adaptive immune response, in particular of T lymphocytes.^54^ Their role in fracture healing can be both detrimental, as well as beneficial, depending on the T cell subsets recruited.^55,56^ For example, terminally differentiated CD8 + T cells were found to secrete pro-inflammatory signals such as TNF-α and IFN-γ in the fracture hematoma. These signals are known to negatively affect MSC osteogenic differentiation in vitro.^55^ Accordingly, depleting CD8 + T cells from an osteotomy gap improved bone regeneration.^55^ Furthermore, Toben et al.^57^ reported faster bone regeneration, lower levels of TNF-α and higher levels of anti-inflammatory cytokines like IL-10, in RAG-1^−/−^ mice model, which lacks an adaptive immune system.^57^ However, when depleting all activated T cells by injecting an anti-CD25 antibody during the inflammatory phase, no improved fracture healing was reported.^58^ This was attributed to the fact that anti-CD25 antibody also depletes regulatory T cells (Tregs), which can promote bone formation through the downregulation of TNF-α and IFN-γ and the secretion of IL-4, a chemoattractant for osteoblast.^51,58^

Even if the initial inflammatory response is a crucial step and initiates the cascade, the resolution of the hematoma and its conversion to granulation tissue is essential for the healing of the fracture. In the subsequent proliferative phase, macrophages, which are known to be an extremely plastic population, acquire mostly an anti-inflammatory and angiogenic phenotype (M2) in response to a change in the surrounding cellular and cytokine milieu.^54^ In particular, M2 macrophages start to secrete VEGF to enhance vascularization in the fracture area^59^ and immunomodulatory cytokines including IL-10 and transforming growth factor β (TGF-β).^54^ TGF-β plays a vital role in chondrogenic differentiation of mesenchymal stem cells for the formation of the soft, cartilaginous callus.^54^ Thereafter, the acquisition of the hypertrophic phenotype is essential for the subsequent mineralization of the callus and its conversion into bone by the joint actions of osteoblasts and osteoclasts.^2^ The newly deposited bone, known as hard callus, is typically irregular. Its remodeling into cortical and/or trabecular bone represents the last stage of the fracture repair.^42^

### Crosstalk between immune cells and bone remodeling

Besides their role in removing dead tissue remnants and in reducing the spread of infection, cells from the adaptive and innate immune systems also affect bone homeostasis.^27,60^ The most obvious immune cells that affect bone homeostasis are the osteoclasts that, like dendritic cells (DC) and macrophages, derive from a myeloid precursor.^27^ Osteoclasts are responsible for the catabolic phase of bone remodeling, which means that they play an active role in bone resorption. Their activity is tightly coupled with the anabolic phase of bone remodeling, where osteoblasts are responsible for new bone deposition.^27^ Key molecules responsible for the connection between osteoclasts and osteoblasts are the receptor activator of nuclear factor κB (RANK), receptor activator of nuclear factor κB ligand (RANKL), and osteoprotegerin (OPG). Their interaction is known as the RANK/RANKL/OPG axis.^27,61,62^ Specifically, RANKL is a transmembrane protein synthesized by the osteoblasts that is involved in osteoclast maturation and activation. Its action is mediated by the binding to its receptor RANK, present on the pre-osteoclast surface.^63^ The balance between bone resorption and deposition is tightly regulated by the presence of OPG, a decoy receptor also secreted by the osteoblasts.^62^ The production of RANKL by several immune cells including monocytes, neutrophils, DC, and B and T lymphocytes, highlights their role in the regulation of osteoclast and osteoblast activity.^62^ Besides RANKL production by multiple immune cells, several more examples can show the tight connection between immune cells and bone homeostasis. For instance, activated T cells can both positively and negatively influence bone homeostasis by secreting osteoclastogenic cytokines, depending on the T cell subpopulation involved.^61^ T helper 17 (Th17) cells, for example, represent the T lymphocyte subpopulation renowned for the involvement in bone resorption. They secrete IL-17 that, besides being a potent stimulator of RANKL expression, induces the synthesis of matrix-degrading enzymes.^62,64^ On the other hand, T helper 1 (Th1) and 2 (Th2) primarily inhibit osteoclasts maturation through the secretion of IFN-γ and IL-4, respectively.^65^ Similarly, regulatory T cells (Tregs) are known to express anti-inflammatory cytokines like IL-4, IL-10, and TGF-β, which suppress ostoclastogenesis.^62^ Besides T lymphocytes, other immune cells are known to be able to influence bone homeostasis. B cells, and in particular bone marrow plasma cells, are known to be involved in modulating the balance between bone resorption and deposition, as they represent a major source of OPG.^61,66^ In addition to adaptive immune cells, innate immune cells, including macrophages and neutrophils, can influence bone formation secreting pro or anti-inflammatory cytokines.^67^ In particular, pro-inflammatory cytokines like TNF-α, IL-6, and IL-1β promote RANKL secretion, increase osteoclast differentiation and resorption capacity, while inhibiting osteoblast differentiation and activity.^27^ On the other hand, anti-inflammatory cytokines including IL-4 and IL-10 increase bone formation by inducing osteoblast proliferation and inhibiting osteoclastogenesis.^62^ Thus, it is clear that immune cells play a role in bone remodeling and the outcome depend on the balance between factors that promote or inhibit osteoclasts maturation and catabolic activity and factors that attract and promote osteoblasts differentiation and bone formation.

## Tissue engineering: balancing the immune response and bone formation

**Fig. 1:**
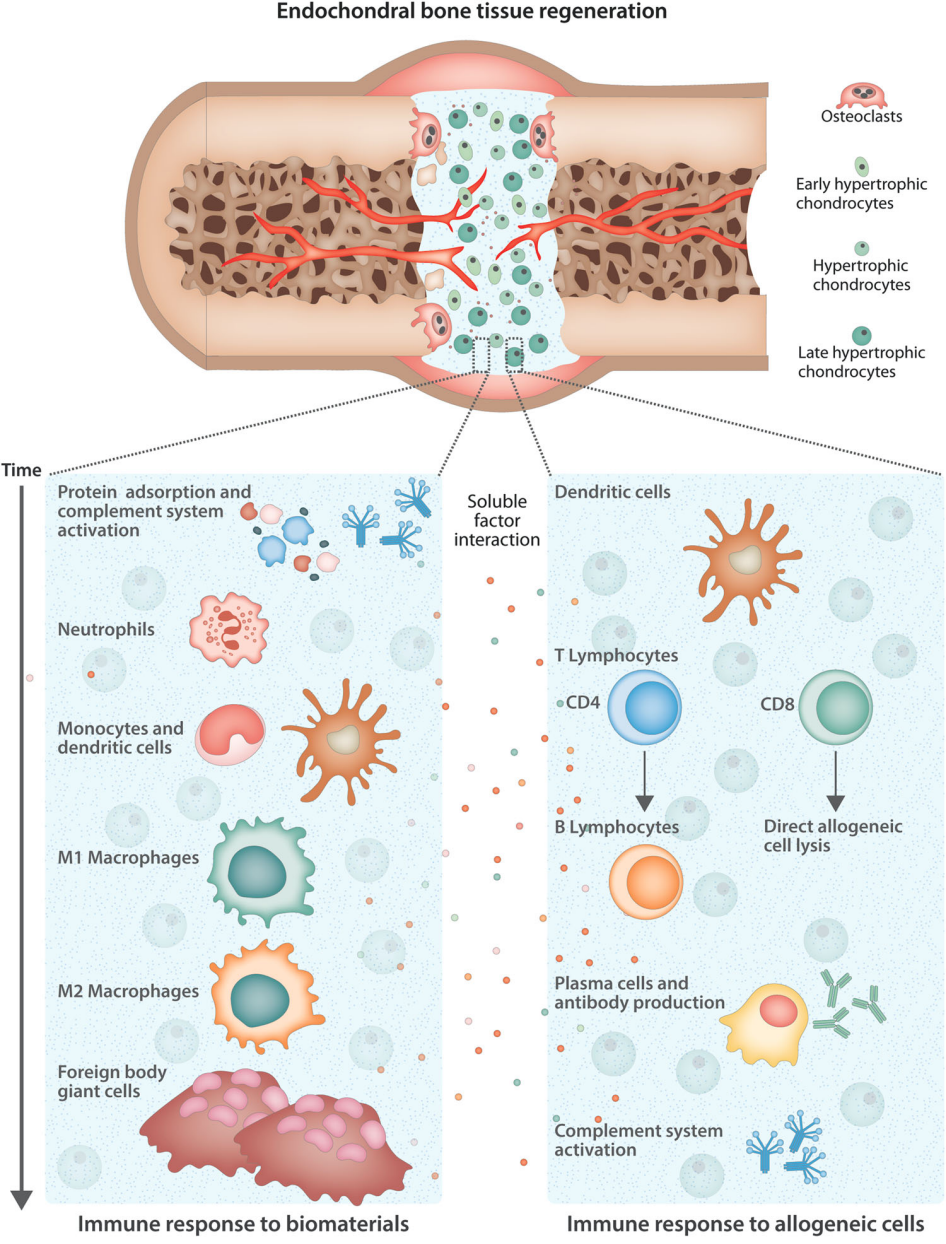
Schematic overview of the cell types involved in the endochondral ossification process induced by an allogeneic tissue engineered construct and the immune response elicited. After implantation, the phenotype of MSC-derived chondrocytes progresses until the late hypertrophic stage, a stage that is characterized by increased secretion of proangiogenic factors and MMPs to promote matrix remodeling and new bone formation. However, the implantation of a biomaterial, together with the presence of allogeneic cells, at the same time induces the recruitment of host immune cells. In particular, the immune response against the carrier biomaterial (left panel) is mainly characterized by the presence of cells from the innate branch of the immune system while the presence of allogeneic cells triggers mostly an adaptive response (right panel). Nonetheless, the recruited cells can influence each other through the engagement of common players (e.g., dendritic cells and the complement system) and through the secretion of soluble factors such as cytokines that can promote the induction of a pro-inflammatory or anti-inflammatory environment. The final outcome of the bone regeneration process is determined by the balance between the promotion of endochondral ossification and the exacerbation of the immune response by the allogeneic construct

Tissue engineered bone substitutes can have various compositions. In general, they can contain biomaterials and/or cells and proteins. The cell source can be either autologous or non-autologous, which includes xenogeneic or allogeneic sources. Frequently, cells used for bone regeneration in preclinical studies are chondrogenically or osteogenically differentiated or undifferentiated MSCs, whether or not incorporated in a scaffold material as a carrier. Such scaffolds are most commonly made of nature-derived materials, such as collagen and fibrin or (semi-)synthetic (bio)polymers, such as poly(e-caprolactone).^68^ Following implantation, the presence of a biomaterial and/or non-autologous cells often can intensify the inflammatory response and eventually affect the outcome of the fracture healing process.^69^ Here, the effects of implantation of biomaterials and allogeneic cells on the immune response and bone formation will be discussed (Fig. 1).

### Immune response to biomaterials

Due to the surgical procedure required for the implantation of the biomaterial, the integrity of the tissue inevitably becomes compromised. In particular, the cell death by necrosis can lead to the release of danger signals known as alarmins (e.g., heat shock proteins, high-mobility-group box proteins and ATP), which can recruit to the implantation site DCs and macrophages.^70^ As a consequence, even if the biomaterial is defined as biocompatible, the implantation itself can trigger an immune response that affects the fracture healing process.^70^ After the implantation, the first step in the cascade of inflammatory events is the activation of the coagulation cascade and the complement system.^70^ The activation of factor XII, the initiator of the intrinsic coagulation cascade, is promoted by its direct contact with the surface of biomaterials^71^ and by platelet adhesion to the surface and activation.^72^ Downstream, thrombin activation catalyses fibrinogen cleavage, to form the primary fibrous mesh around the biomaterial.^70^ The complement system is also activated, mostly via the classical and the alternative pathway.^73^ Together with fibrinogen,^74^ fibrin and the anaphylatoxins of the complement cascade, other proteins adsorb to the biomaterial surface. Among those, fibronectin and vitronectin have a pivotal role in the regulation of the inflammatory response to the implanted biomaterial.^70^ The proteins adsorbed to the surface form a provisional matrix, which influences the subsequent immune cell adhesion and activation.^75,76^ Furthermore, the newly formed matrix is a rich source of chemokines, cytokines, and growth factors involved in attracting immune cells.^77^ Thus, immune cells migration to the implantation site, adhesion and activation on biomaterials mainly occurs through the interaction of adhesion receptors, like integrins with the adsorbed proteins.

Similarly to the fracture healing process, the first cells recruited to the implant site are neutrophils.^77,78^ The interaction with the adsorbed matrix proteins promotes their phagocytic activity, the release of granules loaded with proteases and the production of reactive oxygen intermediates (ROIs).^79^ Together, these destructive agents may damage the implant^80^ and promote the recruitment of monocytes and macrophages.^77^ The infiltration of macrophages and lymphocyte to the implantation site mark the transition from acute to chronic inflammation.^81^ M1 is the macrophage phenotype that is predominantly present during the first stages of inflammation, as these macrophages are directly involved in pathogen killing, secretion of pro-inflammatory cytokines, and Th1 cell recruitment. The uptake of wound debris and apoptotic neutrophils by macrophages can stimulate the production of immunomodulatory molecules, including TGF-β, IL-10, and prostaglandin E2. Together with IL-4 and IL-13 that are secreted by granulocytes, mast cells, and Th2 cells, these immunomodulatory molecules trigger M2 polarization of the macrophages. Depending on the specific M2 macrophage subtype that is being generated, they could be involved in immunomodulation or in tissue repair.^82^ In general, M2 macrophages support wound healing by secreting growth factors like TGF-β, basic fibroblast growth factor (bFGF), platelet-derived growth factor (PDGF), and VEGF, which are involved fibroblast recruitment, proliferation, extracellular matrix (ECM) synthesis, and blood vessel invasion.^70,77^ However, if macrophages fail to phagocytose the biomaterial due to the high material-to-cell size ratio, they fuse together to form foreign body giant cells (FBGCs). If also FBGCs fail in phagocytosing the foreign material, they become frustrated multinucleated macrophages. This means that they increase their degradative capacity, organizing podosomal structures to seal the interface with the biomaterial surface and start to secrete ROIs and degradative enzymes.^70,83^ Interestingly, FBGCs are also thought to be responsible for the secretion of anti-inflammatory cytokines and pro-fibrotic growth factors (e.g., TGF-β and PDGF).^70^ However, continuous action of FBGCs is associated with prolonged fibroblast activation and impaired matrix deposition. In particular, within two to four weeks, the foreign material is encapsulated within an almost avascular, fibrotic tissue capsule, which might lead to the loss of implant function.^84^

The crosstalk between the innate immune response and T lymphocytes is mainly mediated by antigen presenting cells (APCs), in particular by DCs.^85^ According to the type of pathogen recognition receptors (PRRs) involved in the interaction with the biomaterial, different DC maturation stages are stimulated. Immature and semi-matured DCs, for example, stimulate tolerance and limit the inflammatory response whereas fully mature DCs promote the development of an immune response.^70^ The presence of T lymphocytes during the inflammatory phase of the foreign body response directed against an implanted biomaterial has been confirmed in several in vivo studies. The specific T cell subpopulations present in this phase can steer macrophage polarization and fusion.^82^ On the contrary, little is known about lymphocytes B role during immune response against synthetic materials. However, their role becomes crucial when nature-derived biomaterials, such as decellularised tissues, are implanted. In particular, when a biomaterial is derived from non-human primates (e.g., pigs), two different types of antibodies can be produced. The most abundant ones are antibodies against a carbohydrate antigen called “α-gal epitope”, which is present on glycolipids, glycoproteins, and proteoglycans of the ECM. The second type of antibodies, defined as anti-non gal antibodies, is instead produced against different immunogenic peptides of the ECM.^86^

### Immune response to allogeneic cells

Compared to the immune response to biomaterials, the one directed against allogeneic cells is characterized by a more pronounced adaptive component.^69^ The major histocompatibility complex (MHC) molecules, cell-surface glycoproteins known in humans as the human leukocyte antigen (HLA) molecules, represent the principal target of the allogeneic immune response against grafted cells.^87^ Two different classes of MHC, MHC class I and class II, are responsible for the antigen presentation to the T lymphocytes. To present the allogeneic cell antigens to the immune cells, both MHC molecules bind small peptide fragments and display them on the cell surface. Together, the complex of a loaded peptide and a MHC molecule can be recognized by the T cell receptor (TCR). The capability of the TCR to recognize a unique combination of features of both, the loaded peptide and the MHC presenting molecules is known as MHC restriction.^88^ MHC class I and II are characterized by a different structure and distribution among the cells. This affects the type of effector T cells they can interact with. In general, MHC class I, which is present in all nucleated cells, is loaded with intracellular peptides and it is recognised by CD8 + cells. MHC class II is instead loaded with extracellular peptides. It is present on APCs, including macrophages, DC and B lymphocytes and it is recognised by CD4 + T lymphocytes.^89^ However, APCs can also present on their MHC class I extracellular antigens acquired via phagocytosis and endocytosis, in order to activate CD8 + lymphocytes, by a mechanism called cross-presentation or cross-priming.^90^ Considering the complexity and the high polymorphism of the MHC loci, the region of MHC interaction with the TCR and the peptide binding site can differ from one individual to another. As a consequence, when donor cells are implanted, they can be directly recognized by the host immune system because of the differences in peptides presented on the donor MHC and the distinct features of donor MHC molecules.^88^ It has been estimated that a high proportion of 1 to 10% of all mature host T cells will respond to stimulation by cells from another, unrelated member of the same species.^91^ Besides the direct recognition of the foreign cells by T lymphocytes, MHCs from donor cells can be taken up by the host APCs, processed to obtain allopeptides and can be indirectly presented on the APC surface to recipient T cells.^87,92,93^ Further, donor MHC class I and II molecules can be transferred to the host’s APCs via direct cell-to-cell contact or via the release and uptake of exosomes.^94^ As a consequence, it is possible to find host APCs presenting both, allogeneic antigens retrieved by phagocytosis on their MHC class II and also donor MHC class I surface expression derived from the concurrent vesicle trafficking.^92^ These different pathways of allo-recognition are non-mutually exclusive and they all trigger host adaptive immune reactions.^93^ Together this means that, in case of cell transplantation between non-identical or MHC-mismatched individuals, the likelihood of MHC associated rejection is high.^87,95^ However, many factors, including the type of implanted cells, the site of the body where they are introduced and the immunological status of the recipient, can influence the nature and magnitude of the T cell response induced. Furthermore, the ratio of CD4 + and CD8 + T lymphocytes that are activated during the response against the allogeneic antigens can change according to the players involved in the recognition process (e.g., direct recognition of the allogeneic MHC-peptide complex from T cells or indirect activation via indirect presentation by the APC of the host).^96^ Activated CD8 + T cells secrete pro-inflammatory cytokines, including IFN-γ, that promote the skewing of CD4 + T cells toward the pro-inflammatory Th1 cells. Furthermore, both CD8 + and Th1 lymphocytes are responsible for the direct lysis of donor cells.^87^ On the other hand, Th2 lymphocytes secrete interleukins IL-4, IL-5, IL-9, IL 10, and IL-13, involved in the recruitment and activation of eosinophils.^87^ After activation, eosinophils are known to release granules containing enzymes responsible for tissue damage and graft rejection.^87^ In addition to the above mentioned consequences of Th1 and Th2 polarization, CD4 + cells can also establish interactions with B lymphocytes, which can produce anti-MHC class I and II antibodies.^87^ The antibodies produced against the allogeneic antigens will coat the grafted cells, promoting their killing in several ways, including their direct lysis due to the activation of the complement cascade and the natural killer (NK) cells. Besides producing alloantibodies, B cells are also involved in the activation and modulation of T cells, since they are directly involved in antigen presentation. Further, they are involved in modulating the immune response, secreting cytokines like IL-10 and TGB-β.^97^

As previously mentioned, cells from the adaptive immunity are the principal mediator of the allogeneic response. However, recent studies have attributed more importance to the innate branch of the immune system. In particular, since macrophages and neutrophils are the first cells recruited to the site of cell implantation, they can influence lymphocyte activation and polarization through cytokine secretion, promoting eventual rejection or tolerance of the implanted cells.^98^ Furthermore, NK cells are directly involved in allogeneic MHC recognition and in transplanted cells depletion.^98^

## Immune reactions against allogeneic, chondrogenically differentiated MSCs

When implanting cell-seeded constructs for EBR, it is essential that the cells survive in the defect site long enough to initiate the conversion of cartilage into bone. The hematoma microenvironment, as well as the persistent actions of immune cells might act to destroy the grafted MSC-derived chondrocytes prior to the beginning of bone formation.

Culture-expanded MSCs have been shown to exhibit immunomodulatory properties.^99–101^ In particular, they express intermediate to low levels of MHC class I molecules, low levels of co-stimulatory CD40, CD80, and CD86 and very low to no expression of MHC class II, which enables them to evade the immune surveillance by the CD4 + T cells.^100,102^ MSCs have also been shown to inhibit T cell proliferation through indoleamine 2,3- dioxygenase (IDO) and cyclooxygenase-2 (Cox-2) mediated depletion of tryptophan and production of prostaglandin E2 (PGE2), respectively.^103,104^

In addition, MSCs can shift the Th cell phenotype from pro-inflammatory Th1 and Th17 cells to the regulatory Treg phenotype, either by directly influencing their polarization by secreting TGF-β or by inhibiting the proliferation of the inflammatory subsets.^99,104,105^ Besides influencing T cells, MSCs can also play a role in modulating other immune cell types. For example, MSCs can also inhibit DC maturation, resulting in decreased expression of MHC class II and co-stimulatory molecules on DCs surface.^106,107^ Furthermore, they can inhibit B lymphocytes and NK cells activation and expansion through the secretion of TGF-β. Finally, by producing IDO and PGE2, MSCs induce the macrophage skewing toward the anti-inflammatory M2 phenotype.^108^ These mechanisms make undifferentiated MSCs an attractive source in non-autologous transplantation. In particular, recent clinical studies have confirmed the safety of implanting allogeneic MSCs and their beneficial effect in diseases, such as graft versus host disease and Crohn’s disease.^109–111^

However, regenerative constructs that aim to induce EBR are typically seeded with chondrogenically differentiated MSCs. Only a limited number of studies have studied the change in immunomodulatory properties upon chondrogenic differentiation of MSCs so far.^102,112–119^ In 2003, Le Blanc et al.^102^ analyzed the changes in HLA I and II expression when MSCs were differentiated toward the chondrogenic lineage.^102^ Similarly to undifferentiated MSCs, chondrogenically differentiated MSCs expressed intermediate levels of HLA class I and no HLA class II molecules. After stimulating chondrogenically differentiated MSCs with IFN-γ, a low expression of HLA class II was detected, like in undifferentiated MSCs. Further, they showed that chondrogenically differentiated MSCs do not stimulate allogeneic lymphocytes proliferation in co-culture experiments, suggesting the preservation of their capability to not elicit an immune response.^102^ Interestingly, chondrogenically differentiated MSCs also maintain their ability to actively suppress lymphocytes allo-response, indicating that they possess immunosuppressive properties similar to those of undifferentiated MSCs.^102^ Further data supporting the immunomodulatory effect of chondrogenically differentiated MSCs are reported in an in vitro study performed by Zheng et al.^114^ Similarly to Le Blanc et al.^102^ they showed that both undifferentiated and chondrogenically differentiated MSCs can inhibit proliferation and activation of allogeneic T cells.^114^ Additionally, both chondrogenically differentiated and undifferentiated MSCs were equally effective in inhibiting IFN-γ and TNF-α secretion when co-cultured with allogeneic CD4 + and CD8 + T cells, while upregulating the levels of IL-10.^114^ In line with these results, Du et al.^117^ showed that even in a pro-inflammatory environment, MSC-derived chondrocytes displayed immunosuppressive effects on allogeneic T cell proliferation and natural killer cell-mediated cytotoxicity in vitro.^117^ Despite the incomplete understanding of the mechanisms with which chondrogenically differentiated MSCs retain their immunoregulatory properties, it has been proposed that TGF-β1 and HLA-G could play a role herein.^114,117^ It was also suggested that, similar to undifferentiated MSCs,^120^ the presence of allogeneic, chondrogenically MSCs-derived chondrocytes do not induce DC maturation in vitro.^121^ In particular, no upregulation of maturation markers, such as CD80, CD86, and HLA-DR, was observed on DCs during co-culture of chondrogenically differentiated MSCs and immature or LPS-matured DCs. Further, though the DCs infiltrated the chondrogenically differentiated MSC pellets, the presence of chondrogenically differentiated MSCs did not induced an increase in antigen uptake over time.^121^ Taking the aforementioned into account, we could speculate that, even in an in vivo setting, the implantation of allogeneic, chondrogenically differentiated MSCs will not trigger an allogeneic T cell response. Furthermore, even if a T cell response would be triggered, an active suppression is expected. The formation of a tolerogenic environment would allow the remodeling of the cartilaginous construct into new bone tissue, following the endochondral pathway.

However, results that contrast the above observations have been reported in literature. A recent study by Kiernan et al.^115^ demonstrated that chondrogenically differentiated MSCs were not able to actively alter the proliferation of allogeneic CD4 + and CD8 + T cells in vitro. Further, histological analysis revealed that after co-culturing peripheral blood mononuclear cells with chondrogenically differentiated MSCs in vitro, T cells (as identified using anti-CD3 antibodies) infiltrated the chondrogenic matrix. Although in this study these infiltrating lymphocytes did not appear active, as they showed low expression levels of genes coding for T activation proteins, including CD25 and CD69, and pro-inflammatory cytokines like TNF-α^115^ Chen et al.^113^ reported the ability of chondrogenically differentiated MSCs to stimulate lymphocyte proliferation, cytotoxicity and DC maturation in vitro. The authors suggested that the upregulation of the co-stimulatory molecules CD80 and CD86 on the chondrogenically differentiated MSCs could be involved in this response, since blocking their expression reduced DCs maturation and restored levels of T lymphocytes proliferation similar to the ones of the undifferentiated MSCs.^113^ Even though this study^113^ was performed in a xenogeneic setting, as rat MSCs were presented to human DC, the loss of immunosuppressive properties in MSC-derived chondrocytes was confirmed in an allogeneic setting, both in vitro and in vivo.^112^ In particular, undifferentiated or chondrogenically differentiated MSCs were implanted subcutaneously alone or in combination with an alginate gel in a fully MHC-mismatched allogeneic rat model. Higher number of CD3 + lymphocytes and CD68 + macrophages infiltrated the alginate carrier when chondrogenically differentiated MSCs were encapsulated. Further, T cells reactive to allogeneic antigens were found in the draining lymph node of both, the rat group in which differentiated MSCs were implanted, as well as in the one that received undifferentiated MSCs. After the encapsulation of the allogeneic cells into an alginate carrier, a protective effect was observed for the undifferentiated MSCs group, as no antidonor T cell response was observed in the local lymph node. However, such positive influence was not observed in the chondrogenically differentiated group. Finally, it must be noted that, despite its protective role against T cells, the encapsulation in alginate enhanced the production of donor-specific IgG_2_ antibodies.^112^ Similar results were obtained by Butnariu-Epharat et al.^122^ in an orthotopic implantation model in goats. In particular, when allogeneic, chondrogenically differentiated MSCs where embedded in a hyaluronic acid-based gel to resurface the articular knee cartilage, a mild immunologic rejection characterized by blood cell infiltrates was observed. These findings offer valuable insight in the differences in immune reaction to differentiated and undifferentiated MSCs, as well as the role of an encapsulating biomaterial in vivo.

The contrasting in vitro results and the scarceness of in vivo studies do not provide a clear portrait of the immunological processes associated with allogeneic chondrogenic MSC implantation.^116,119^ The contradictory results could be partially explained by the differences in the ratios of chondrogenically differentiated MSCs and T cells, as it has been show that in vitro, the suppressive action of the MSC-derived chondrocytes on T cell activation is dose dependent.^114,115,117^ More specifically, Ryan et al.^112^ observed an immune response to differentiated MSCs using a low MSC/T ratio (1:50 and 1:100) compared to the studies carried out by Kiernan et al.^115^ and Zheng et al^114^ (1:5 and 1:1, 1:5 and 1:10, respectively). Further, the composition of the induction medium, as well as the culture and assay conditions might play a role in increasing the immunogenicity of chondrogenically differentiated MSCs.^117,118,123^ For example, TGF-β, a key component of chondrogenic induction medium, is also involved in regulation of the expression of HLA-DR^124^ and CD80 and CD86.^113^ This should be taken into account, especially when the changes in expression of these molecules is analyzed. Finally, it must be noted that most of the studies are focusing only on T lymphocytes response, whereas little is known about allogeneic, chondrogenically differentiated MSCs influence on DCs, macrophages, NK cells, and B cells. Thus, so far it is not possible to define whether MSCs-derived chondrocytes will evoke an immune response upon implantation. Further, the possible interference of a potential allogeneic response with the endochondral process remains yet to be elucidated.

## Implications of immune reactions for EBR

In the previous sections, the physiological healing process and its alteration due to the immune response induced by the implantation of a biomaterial and allogeneic, chondrogenically differentiated MSCs were discussed. Only few studies have studied the changes of immunological properties of allogeneic MSCs-derived chondrocytes in vitro or in vivo. However, a great number of studies have evaluated the immunogenicity of allogeneic chondrocytes for cartilage tissue engineering. Thus, in this section we will try to integrate this information and speculate on what could be expected after the implantation of an engineered cartilage template in terms of its endochondral bone regenerative capacity.

In EBR, chondrocytes within the cartilage template acquire a hypertrophic phenotype and start to secrete proangiogenic factors and metalloproteinases to promote blood vessel invasion and matrix remodeling. As a result, host cells involved in bone remodeling are recruited to the implantation site and promote cartilage remodeling and new bone formation.^4^ This process could potentially be hampered by an abnormal chronic inflammation induced by both the carrier biomaterial and the MSC-derived chondrocytes.

As described in the previous section, an increased immunogenicity of allogeneic, chondrogenically differentiated MSCs was observed in vivo.^112,122^ Although similar results were sometimes obtained when implanting allogeneic chondrocytes,^125–127^ an overwhelming number of in vitro and in vivo studies suggest that cartilage tissue possesses immunoprivileged properties, and no, or only a minor immune response is elicited in an allogeneic setting.^125,128–132^ This is thought to be due to the presence of a tight extracellular matrix that shields chondrocyte-associated antigens and protects the embedded cells from the immune surveillance.^125^ Thus, the discrepancy between these results could be at least partially explained by heterogeneity in MSCs chondrogenic differentiation.^122,125^ The reduced ECM shielding due to the retention of a small, undifferentiated MSCs subpopulation might have hampered the immunoprivileged characteristics of cartilage, enhancing host immune reaction. Even though the main goal of the studies involving allogeneic chondrocytes was to obtain stable articular cartilage and not EBR, these results tend to support the idea that only a minor immune reaction against an engineered, allogeneic cartilaginous construct is to be expected. Therefore, even though evidence is diverging, we postulate that EBR will not be hampered in its first steps because of a reaction against the cartilage template and could proceed toward the remodeling phase. After the conversion from cartilage to bone, understanding the fate of the chondrocyte is crucial to define the time span within which the host is exposed to the allogeneic antigens. It has long been accepted that the endochondral processes encompassed chondrocyte terminal differentiation^133^ including apoptosis.^134–136^ As a result, allogeneic cells in EBR would eventually disappear from the implantation site and the allogeneic cells and antigens would only be exposed to the immune system for a limited amount of time. However, outcomes of recent studies in lineage tracing support the hypothesis that only a subset of the hypertrophic chondrocytes undergo apoptosis, while most transdifferentiate to osteoblasts and osteocytes.^48,134,137,138^ The mechanisms at the base of this transformation to osteogenic cells are not fully understood yet.^134^ Nevertheless, the implications for EBR are evident. At least part of the regenerated bone would be donor-derived, which means that the allogeneic cells and antigens could be exposed to the host immune system cells for a longer period.^48^ Several studies describe the immunogenicity of allogeneic, osteogenically differentiated MSCs.^26,116,119^ Overall, it seems that allogeneic MSC-derived osteoblasts induce a milder allogeneic immune reaction compared to the chondrogenically differentiated MSCs both in vitro^102,113,139–141^ and in vivo.^33,34^ In particular, osteogenically differentiated MSCs seem to retain immunoevasive and immunomodulatory properties similar to the undifferentiated MSCs, since they not only fail to stimulate alloreactive lymphocytes responses, but they also actively suppress T cells proliferation in vitro.^102^ Further, MSC-derived osteoblasts showed an inhibitory effect on DCs maturation even in a xenogeneic setting.^113^ However, the implications of the presence of these allogeneic, osteogenic cells in the bone regenerative process are unknown.

The intricacy of the interactions between host immune cells, implanted cells and the carrier material, complicates a reliable prediction of their effect on the EBR process. The low immunogenicity of the cartilage matrix, together with the suppressive effect on T lymphocytes of MSC-derived chondrocytes support the feasibility of using allogeneic, chondrogenically differentiated MSCs for endochondral bone tissue engineering applications. However, these immunoevasive and immunomodulatory properties might change during the cartilaginous template remodeling, as the blood vessels invasion required for EBR could disrupt the protective ECM shield, exposing the allogeneic MSC-derived chondrocytes. Thus, to investigate the complex balance between EBR and immune response the use of a relevant preclinical animal model is required (as discussed in ref. ^119^).

## Conclusive remarks and future perspective

The implantation of an allogeneic MSC-containing engineered construct for EBR purposes will alter the inflammatory phase of the fracture healing process due to the presence of a biomaterial and the chondrogenically differentiated, allogeneic cells. Depending on the balance between pro- and anti-inflammatory cytokines and immune cell subsets, the survival of the construct and the regeneration process may be hampered or improved.

To reduce the chances of developing a strong immune response, selecting the appropriate carrier material is of pivotal interest. Firstly, the biomaterial should support MSCs chondrogenic differentiation and offer shielding from the immune system. In addition, the use of immunomodulating biomaterials represents a promising strategy to tailor the immune cell recruitment, enhancing bone healing and promoting the integration of the cell-seeded constructs. In particular, changing the surface chemistry will influence protein adsorption on the biomaterial surface, dictating the type of immune cell that will interact with it. Alternatively, the incorporation of bioactive molecules can induce the creation of a more tolerogenic environment, which will prevent the implant rejection.^70^ In particular, the local delivery of immunosuppressants through their incorporation into the carrier material could represent a promising strategy^142,143^ as the temporary release of the drugs could buy enough time for the allogeneic cartilage template to remodel into new bone tissue, avoiding the complications associated with systemic immune suppression.

Considering the use of an allogeneic cell source, a (partially) HLA matched donor might help in reducing the immune reaction against the differentiated chondrocytes or osteocytes. In particular, a higher transplant success rate after 10 years from the surgery has been reported for kidney transplantation when HLA are fully, or at least partially matched between donor and patient.^144^ It has been shown that the impact in graft loss depends mostly on the effects of three antigens, HLA-A, HLA-B, and HLA-DR. In particular, the impact of an HLA-DR mismatch can be observed in the first 6 months after transplantation, whereas the HLA-B mismatch effect emerges in the first 2 years, and HLA-A mismatches have an adverse effect on long-term in renal graft survival.^95,145^ This may suggest that, for EBR applications it might be enough to have an HLA-DR and/or HLA-B match, since part of the allogeneic cells will be lost during the cartilage conversion into bone and the newly formed bone tissue will be remodeled and slowly replaced entirely by the host tissue. This means that, by the time HLA-A mismatch effects manifest themselves, the donor cells will not be present in the host anymore.

Finally, a logical next step in EBR research entails the development of a clinically relevant immunocompetent animal model to validate the regenerative potential of differentiated allogeneic MSCs. Kovach et al.^60^ reported a discrepancy between immunogenicity of allogeneic MSCs when transplanted into mouse models, compared to larger animal models. In particular, the majority of the studies performed in mice demonstrated immunogenic properties of allografted MSCs, while in larger animals such as rabbits, dogs and sheep this was not the case. In addition, Kovach et al. noticed that the majority of the studies demonstrating the immunoprivileged status of MSCs were performed in a bone orthotopic setting (e.g., bone healing).^60^ This suggested that some factors in the inflammatory environment after a bone injury promote allogeneic MSCs survival and differentiation. Thus, the location of implantation should be carefully chosen when the interaction with the immune system is analyzed.

In conclusion, when aiming at developing tissue engineered constructs for bone regeneration, it is crucial to consider if and in which ways the implanted biomaterials and/or cells could trigger an immune response. Different immune cells involved in the response can promote either bone formation or bone resorption, affecting the regenerative outcome. As an instrumental example, we evaluated the potential immunological effects when implanting allogeneic, chondrogenically differentiated MSCs for bone regeneration. Further steps need to be taken to evaluate whether they represent a realistic option to improve the clinical translation of EBR. Balancing the immune responses with regenerative processes will be a next challenge in this promising field.
